# Hippo Signaling Pathway Dysregulation in Human Huntington’s Disease Brain and Neuronal Stem Cells

**DOI:** 10.1038/s41598-018-29319-4

**Published:** 2018-07-27

**Authors:** Kaly A. Mueller, Kelly E. Glajch, Megan N. Huizenga, Remi A. Wilson, Eric J. Granucci, Amanda M. Dios, Adelaide R. Tousley, Maria Iuliano, Elizabeth Weisman, Michael J. LaQuaglia, Marian DiFiglia, Kimberly Kegel-Gleason, Khashayar Vakili, Ghazaleh Sadri-Vakili

**Affiliations:** 1 NeuroEpigenetics Laboratory, MassGeneral Institute for Neurodegenerative Disease (MIND), Massachusetts General Hospital, Boston, MA 02129-4404 USA; 2 Cellular Neurobiology Laboratory, MassGeneral Institute for Neurodegenerative Disease (MIND), Massachusetts General Hospital, Boston, MA 02129-4404 USA; 3 Department of Surgery, Boston Children’s Hospital, Boston, USA

## Abstract

The Hippo signaling pathway is involved in organ size regulation and tumor suppression. Although inhibition of Hippo leads to tumorigenesis, activation of Hippo may play a role in neurodegeneration. Specifically, activation of the upstream regulator, mammalian sterile 20 (STE20)-like kinase 1 (MST1), reduces activity of the transcriptional co-activator Yes-Associated Protein (YAP), thereby mediating oxidative stress-induced neuronal death. Here, we investigated the possible role of this pathway in Huntington’s disease (HD) pathogenesis. Our results demonstrate a significant increase in phosphorylated MST1, the active form, in post-mortem HD cortex and in the brains of CAG knock-in Hdh^*Q111/Q111*^ mice. YAP nuclear localization was also decreased in HD post-mortem cortex and in neuronal stem cells derived from HD patients. Moreover, there was a significant increase in phosphorylated YAP, the inactive form, in HD post-mortem cortex and in Hdh^*Q111/Q111*^ brain. In addition, YAP was found to interact with huntingtin (Htt) and the chaperone 14-3-3, however this interaction was not altered in the presence of mutant Htt. Lastly, YAP/TEAD interactions and expression of Hippo pathway genes were altered in HD. Together, these results demonstrate that activation of MST1 together with a decrease in nuclear YAP could significantly contribute to transcriptional dysregulation in HD.

## Introduction

Huntington’s disease (HD) is an autosomal dominant fatal neurodegenerative disease characterized by motor, cognitive, and behavioral symptoms marked by cerebral atrophy^1^. The causative mutation in HD is a cytosine-adenosine-guanine (CAG) trinucleotide repeat expansion coding for a polyglutamine (polyQ) moiety in the huntingtin (Htt) protein^2^. Htt is ubiquitously expressed and its interactions with a variety of proteins suggests that mutated Htt interferes with multiple cellular pathways causing mitochondrial dysfunction, aberrant signal transduction, abnormal vesicle trafficking, disruption of the ubiquitin-proteasome, deficits in autophagy, neuronal death, and transcriptional dysregulation, to name a few^3–5^. While extensive studies have identified multiple pathways that are affected by mutant Htt (mHtt) in models of HD as well as in HD patients, no single mechanism has emerged as a central pathogenic mechanism. Thus, the complex pathogenesis together with the lack of validated targets in HD presents a significant challenge for developing therapies.

A key pathogenic mechanism in HD is transcriptional dysregulation^3,6,7^. Alterations in gene expression occur early in HD and have been demonstrated in patients as well as in multiple animal and cellular models of HD. Specifically, HD patients and knock-in transgenic mouse models demonstrate alterations in mRNA expression^6,8^ that encompass changes in neurotransmitter receptor mRNA and protein levels that occur before the phenotypical disease onset^9–12^. These findings are consistent with positron emission tomography (PET) studies which demonstrated that dopamine D1 and D2 receptors are decreased in gene-positive but clinically asymptomatic patients^13,14^. Following these initial studies, microarray analyses demonstrated transcript changes in several mouse and cellular HD models^3,6,10,15^. Overall these findings agree with genome-wide studies from human brain tissue samples^16^ and to a lesser degree with studies analyzing human blood samples^17^. Together these studies highlight that numerous genes are altered in symptomatic HD patients, and more importantly, that these changes are recapitulated in animal models suggesting that transcriptional dysregulation is an important and early process in HD pathogenesis. However, the mechanisms that lead to selective alterations in gene expression remain unclear. Recent studies from our laboratory as well as others have focused on identifying critical and novel transcriptional modifiers that may be involved in HD pathogenesis. One such candidate is the Hippo signaling pathway, specifically, its terminal effector component, the transcriptional activator Yes-Associated Protein (YAP).

Although the Hippo signaling pathway was initially discovered in *Drosophila melanogaster*, it has been implicated in mammalian organ size regulation and tumor suppression by regulating both apoptosis and cell proliferation^18–21^. The nuclear effector of this pathway, YAP, is a transcriptional co-activator that binds to and activates the transcription factor TEAD (transcriptional enhancer activator domain)^19,22,23^. Together, the YAP/TEAD complex promotes transcription of pro-survival genes that stimulate cell survival and proliferation while inhibiting apoptosis^22,24^. Increases in YAP activity are also associated with cell proliferation and cancers in other organs^20,22^. In contrast, increases in the activity of mammalian Ste20-like serine/threonine kinase 1/2 (MST1/2), an upstream regulator of YAP, controls organ size, cell proliferation, and induces apoptosis. Specifically, phosphorylation of MST1/2 (active form) leads to phosphorylation of YAP, its cytoplasmic retention, and subsequent degradation^19,22^. Consequently, once phosphorylated, YAP can no longer translocate to the nucleus, is unable to bind to its transcriptional coactivator TEAD, and loses its transcriptional activity^19,21,23^. Thus, cells with hyperactive MST1/2 signaling undergo apoptosis, a process that is directly linked to decreases in nuclear YAP activity. While MST1/2 signaling is decreased during tumorigenesis and YAP nuclear levels are increased, recent studies have revealed that activation of MST1/2 is linked to neurodegeneration^22,25–28^. In HD, decreases in YAP were linked to transcriptional repression-induced atypical cell death (TRIAD), a morphologically and biologically distinct form of cellular death induced by alterations in transcription^25^, as well as mHtt-induced neuronal death termed ‘ballooning cell death’ (BCD)^26^. Together these studies demonstrate that mHtt disrupts YAP-TEAD interactions and suggest that inhibiting Hippo signaling or increasing YAP nuclear activity may provide a new target for the development of treatments in HD. The current study sought to build on previous findings and to determine if Hippo signaling is dysregulated in post-mortem human HD brain and the mechanisms whereby YAP nuclear activity is altered in HD.

## Results

### YAP subcellular localization in post-mortem human cortex and neuronal stem cells

**Figure 1:**
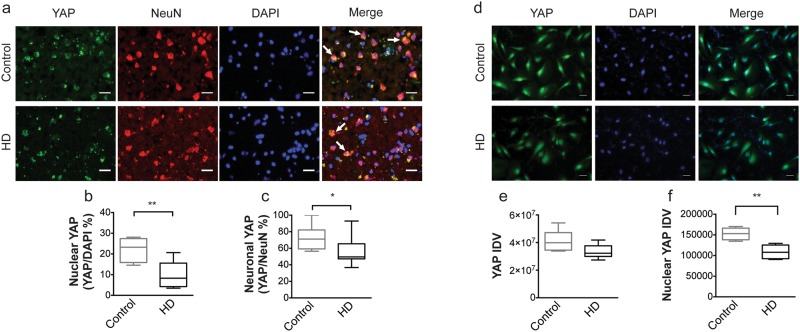
YAP subcellular localization in human post-mortem cortex and NSCs derived from HD patients. (**a**) Representative images from unaffected control (top; n = 7) and HD (bottom; n = 10) human cortical tissue stained with YAP (green), NeuN (red), and DAPI (blue) (left to right). Merged images (far right) show overlap of YAP, NeuN and DAPI. Arrows highlight representative cells; scale bar = 50 μM. (**b**) The percentage of NeuN+ cells that were YAP+ (YAP/NeuN%) was significantly lower (p = 0.0250, Mann-Whitney *U* Test) in HD (black) compared to control (grey) human tissue. (**c**) The percentage of DAPI+ cells that were YAP+ (YAP/DAPI%) was significantly lower (p = 0.0020, Mann-Whitney *U* Test) in HD compared to control tissue. (**d**) Representative images from unaffected control (WAD9) (top; n = 5) and HD (G018) (bottom; n = 6) human embryonic-derived NSCs stained with YAP (green; left) and DAPI (blue; middle). Merged images (right) show overlap of YAP and DAPI. Scale bar = 25 μM. (**e**) Overall YAP intensity was not different (p = 0.1255, Mann-Whitney *U* Test) between control (grey) and HD (black) NSCs. (**f**) Nuclear YAP staining was significantly lower (p = 0.0043, Mann-Whitney *U* Test) in HD compared to control tissue. Data are presented as median and min-max values. *p < 0.05, **p < 0.01.

YAP is transcriptionally active when it is localized to the nucleus^19^. Therefore, we sought to determine whether YAP expression and nuclear localization is altered in HD. To address this, we measured YAP protein levels in neurons and glia using co-localization immunofluorescence in cortical samples from 10 HD patients and 7 controls. Anti-YAP antibody was used together with anti-NeuN (neuronal marker), anti-GFAP (glial marker), and DAPI (nuclear marker). Image J analysis following microscopy demonstrated that there was a significant decrease in YAP levels in neurons, as measured by NeuN co-localization, in HD compared to control (p = 0.0250, Mann-Whitney *U* Test) (Fig. 1a,b). Additionally, there was a significant decrease in YAP nuclear localization in neurons in HD patients compared to control cortex as measured by co-localization with DAPI (p = 0.0020, Mann-Whitney *U* Test) (Fig. 1c). This change was not due to an overall decrease in cell numbers as the total number of NeuN+ cells did not change (p = 0.1689, Mann-Whitney *U* Test) and the total number of DAPI+ cells was increased in HD (p = 0.0172, Mann-Whitney *U* Test) relative to control cortex (Fig. S1). Moreover, there was no change in YAP levels in astrocytes (p > 0.9999, Mann-Whitney *U* Test) as measured by co-localization with the glial marker, GFAP (Fig. S2). To confirm the decreases in neuronal nuclear YAP we performed nuclear and cytoplasmic extractions using the same control and HD cortical samples used in the immunofluorescence experiments. These results indicate that while there is no significant difference in cytoplasmic YAP (p = 0.3095, Mann-Whitney *U* Test), there is a significant decrease in nuclear YAP levels in HD compared to control (p = 0.0152, Mann-Whitney *U* Test) (Fig. S3).

We also assessed YAP expression and localization in neuronal stem cells (NSCs) derived from HD patients using immunofluorescence co-localization. While there was no significant change in total YAP intensity (p = 0.1255, Mann-Whitney *U* Test) (Fig. 1d,e), there was a significant decrease in nuclear YAP (p = 0.0043, Mann-Whitney *U* Test) (Fig. 1d,f) in HD NSCs derived from human embryonic stem cells (GENEA018) compared to unaffected control cell lines (WA09). Although, there was no significant change in total YAP intensity (p > 0.9999, Mann-Whitney *U* Test), there was a trend towards a decrease in nuclear YAP levels (p = 0.0931, Mann-Whitney *U* Test) in iPSC-derived HD NSCs (HD4) compared to control cell lines (WT97) (Fig. S4). Together these results demonstrate that neuronal nuclear YAP levels are decreased in human post-mortem brains as well as NSCs derived from HD patients confirming previous findings^26^.

### YAP mRNA expression in post-mortem human cortex

**Figure 2:**
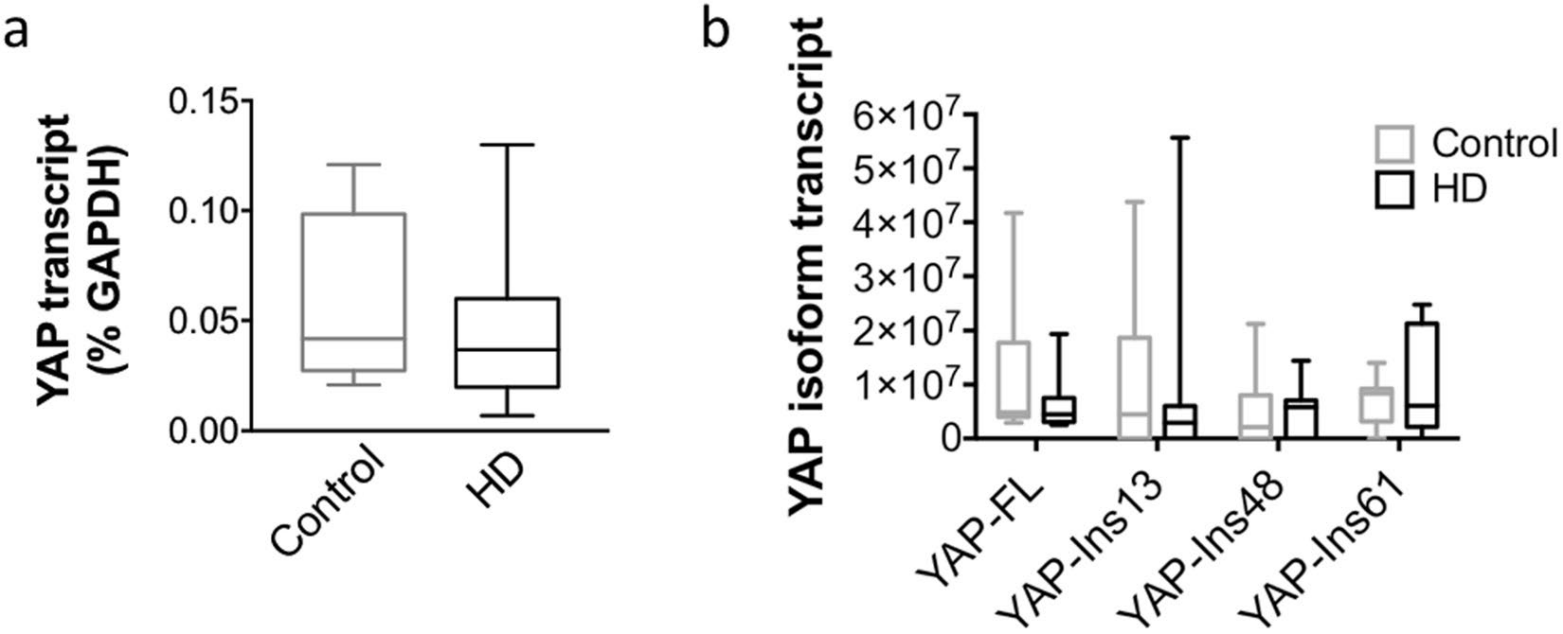
YAP mRNA expression in post-mortem human cortex. (**a**) Quantitative real time PCR assay illustrates no change in YAP mRNA relative to GAPDH levels (p = 0.397, Mann Whitney *U* test) between unaffected control (n = 5; grey) and HD (n = 12; black) patients. (**b**) Quantitative real time PCR assay also demonstrates no change (p > 0.05, Two-way ANOVA) in full length YAP (YAP-FL; left) or YAPΔC isoforms with 13, 48, and 61 nucleotide inserts (left to right: YAP-Ins13, YAP-Ins48, and YAP-Ins61, respectively) between unaffected control and HD patients. A two-way ANOVA demonstrated that there was no significant effect of genotype [F(3, 68) = 0.775, p = 0.512], YAP isoform [F(1, 68) = 0.282, p = 0.597], or interaction [F(3, 68) = 0.383, p = 0.765] in control (n = 8; grey) and HD (n = 11; black) patients. Data are presented as median values.

Given the decrease in neuronal YAP in HD cortex, we sought to determine whether there was a concomitant decrease in YAP transcription. Total RNA was extracted from post-mortem control and HD cortices and assessed for changes in transcript levels using reverse transcription followed by quantitative real time PCR (RT-qPCR) using specific YAP primers as described previously^20^ and normalized to GAPDH. The results indicate that there is no change (p = 0.397, Mann Whitney *U* test) in YAP transcript levels in HD cortex compared to control (Fig. 2a) suggesting that decreases in neuronal YAP protein may be due to an increase in YAP phosphorylation and perhaps degradation as previously described^19,22,29^. We also measured YAPΔCs, novel and previously described neuron-specific YAP isoforms^25^, in the same cortical samples using PCR with the previously published YAPΔC primers^25^. A two-way ANOVA demonstrated that there was no significant effect of genotype [F(3, 68) = 0.779, p = 0.512], YAP isoform [F(1, 68) = 0.282, p = 0.597], or interaction [F(3, 68) = 0.383, p0.765] in 11 HD compared to 8 control samples (Fig. 2b) demonstrating that YAPΔC isoforms are not altered in HD in our post-mortem samples.

### Phosphorylated YAP, MST1/2, and LATS levels in post-mortem human cortex

**Figure 3:**
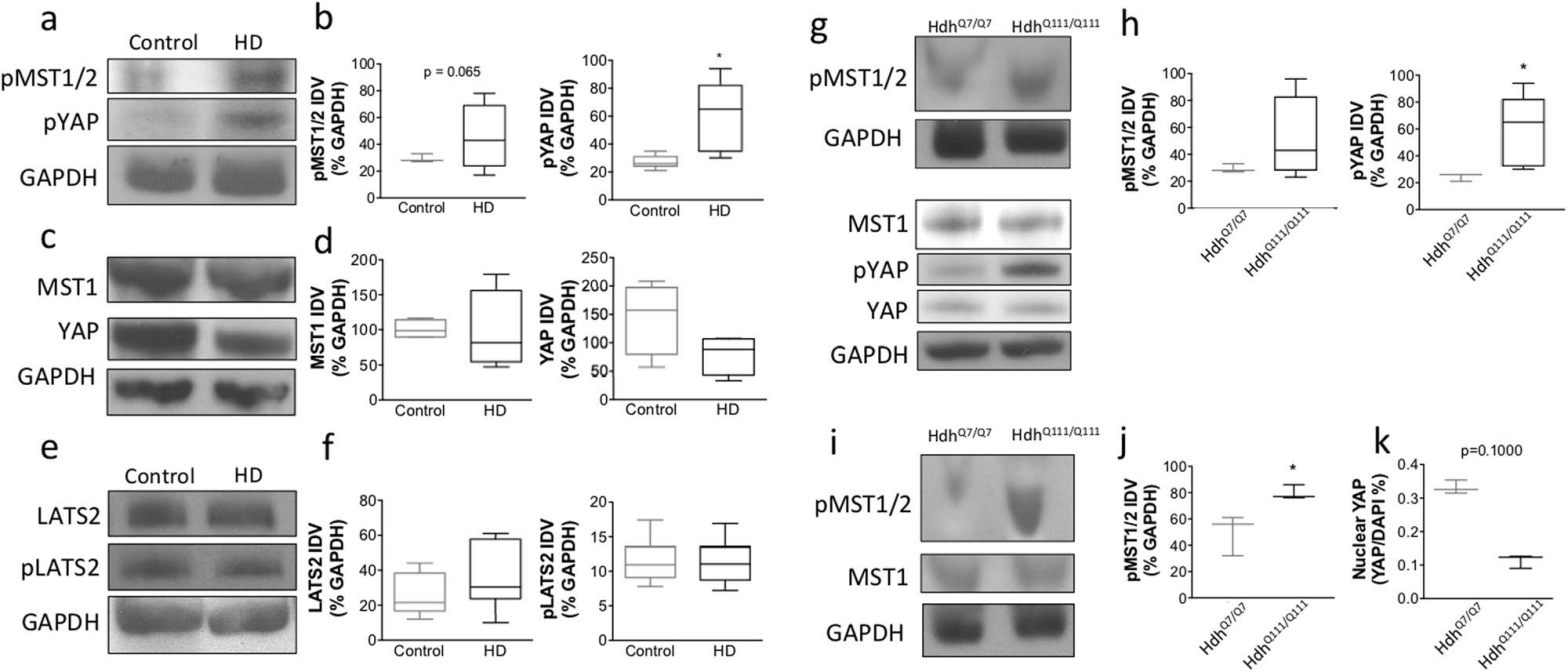
MST1/2 is activated and YAP is inactivated in post-mortem human cortex and the Hdh mouse model of HD. (**a**) Representative western blots for pMST1/2, pYAP and GAPDH (top to bottom) with human cortex samples from unaffected control (left; n = 4) and HD (right; n = 4) patients. (**b**) There was a trend towards an increase (p = 0.065, Mann-Whitney *U* Test) in pMST1/2 levels (left) from HD compared to controls. pYAP protein levels (right) were significantly increased (p = 0.015, Mann-Whitney *U* Test) in HD compared to controls. (**c**) Representative western blots for MST, YAP and GAPDH (top to bottom) with human cortex samples from unaffected control (left; n = 4) and HD (right; n = 4) patients. (**d**) There was no change in MST1 (left) (p = 0.343, Mann-Whitney *U* Test) or YAP (right) (p = 0.200, Mann-Whitney *U* Test) between HD and controls. (**e**) Representative western blots for LATS2, pLATS2 and GAPDH (top to bottom) with human cortex samples from unaffected control (left; n = 8) and HD (right; n = 8) patients. (**f**) There was no change in LATS2 (left) (p = 0.2039, Mann-Whitney *U* Test) or pLATS2 (right) (p = 0.8785, Mann-Whitney *U* Test) in HD compared to controls. (**g**) Representative western blots for phosphorylated MST1/2 (pMST1/2) and GAPDH (top to bottom) from HdhQ7/Q7 (left; n = 3) and HdhQ111/Q111 (right; n = 4) striatum and for MST1, phosphorylated YAP (pYAP), YAP and GAPDH (top to bottom) from Hdh^*Q7/Q7*^ (left; n = 3) and Hdh^*Q111/Q111*^ (right; n = 5) striatum. (**h**) Quantification of westerns blots showed no change in pMST1/2 (left) (p = 0.4000, Mann-Whitney *U* Test) and increased pYAP (right) (p = 0.0357, Mann-Whitney *U* Test) protein levels in Hdh^*Q111/Q111*^ relative to Hdh^*Q7/Q7*^ striatum. (**i**) Representative western blots for pMST1/2, MST1, and GAPDH (top to bottom) from Hdh^*Q7/Q7*^ (left; n = 3) and Hdh^*Q111/Q111*^ (right; n = 4) cortex. (**j**) Quantification of western blots shows increased pMST1/2 (p = 0.0286, Mann-Whitney *U* Test) protein levels in Hdh^*Q111/Q111*^ (right; n = 4) relative to Hdh^*Q7/Q7*^ (left; n = 4) cortex. (**k**) Quantification of immunofluorescence staining illustrated a trend towards a decrease (p = 0.1000, Mann-Whitney *U* Test) in nuclear YAP (YAP/DAPI%) levels in Hdh^*Q111/Q111*^ (n = 3) relative to Hdh^*Q7/Q7*^ (n = 3) samples. Data are presented as median and min-max values. * p < 0.05.

Our data indicate that there is a decrease in nuclear YAP in HD. Since YAP phosphorylation can sequester YAP in the cytoplasm, we assessed alterations in phosphorylated YAP at serine 127 (pYAP) in the same cortical samples using western blots with antibodies specific for pYAP as described previously^18,20^. There was a significant increase in pYAP levels in human HD cortex compared to control (p = 0.015, Mann-Whitney *U* Test) (Fig. 3a,b) but no change in total YAP levels (p = 0.200, Mann-Whitney *U* Test) (Fig. 3c,d). Activation of MST1/2 (pMST1/2) has also been associated with increases in pYAP, therefore total MST1 and pMST1/2 were measured by western blots in the same human cortical samples. While there is no change in total MST1 levels (p = 0.343, Mann-Whitney *U* Test) (Fig. 3c,d), there was a trend towards an increase in pMST1/2 levels in cortical tissue from HD patients compared to controls (p = 0.065, Mann-Whitney *U* Test) (Fig. 3a,b). In addition to activation of MST1/2, LATS activation has been shown to directly increase YAP phosphorylation and thereby lead to sequesteration of YAP in the cytoplasm. Therefore we assessed LATS and pLATS levels given the increase in pYAP in HD. There was no change in LATS2 (p = 0.2039, Mann-Whitney *U* Test) (Fig. 3e,f) or phosphorylated LATS2 (p = 0.8785, Mann-Whitney *U* Test) (Fig. 3e,f) in cortical tissue from HD patients compared to control. These data suggest that while LATS is not altered in HD, increases in pMST1/2 could underlie the increase in YAP phosphorylation and thereby support the significant decreases detected in nuclear YAP levels in HD post-mortem brains.

### Phosphorylated YAP and MST1/2 levels in the Hdh mouse model

Next, we assessed whether similar dysregulation of the Hippo pathway is recapitulated in a mouse model of HD. Similar to the findings in HD patients, while there was no change total YAP (p = 0.571, Mann-Whitney *U* Test), there was a significant increase in pYAP (Fig. 3g,h) in the striatum of 10-month-old Htt CAG knock-in mice Hdh^*Q111/Q111*^ compared to wild-type Hdh^*Q7/Q7*^ (p = 0.0357, Mann-Whitney *U* Test). However, there was no difference in pMST1/2 levels (p = 0.4000, Mann-Whitney *U* Test) (Fig. 3g,h) in Hdh^*Q111/Q111*^ compared to wild-type Hdh^*Q7/Q7*^ striata. In contrast, there was a significant increase in pMST1/2 levels in the cortex of 10-month-old Htt CAG knock-in mice Hdh^*Q111/Q111*^ compared to wild-type Hdh^*Q7/Q7*^ (p = 0.0286, Mann-Whitney *U* Test) (Fig. 3i,j). There was also a trend towards a decrease (p = 0.1000, Mann-Whitney *U* Test) in nuclear YAP levels in Hdh^*Q111/Q111*^ mice (Fig. 3k) similar to previous findings^26^.

### YAP-Htt-14-3-3 interactions

**Figure 4:**
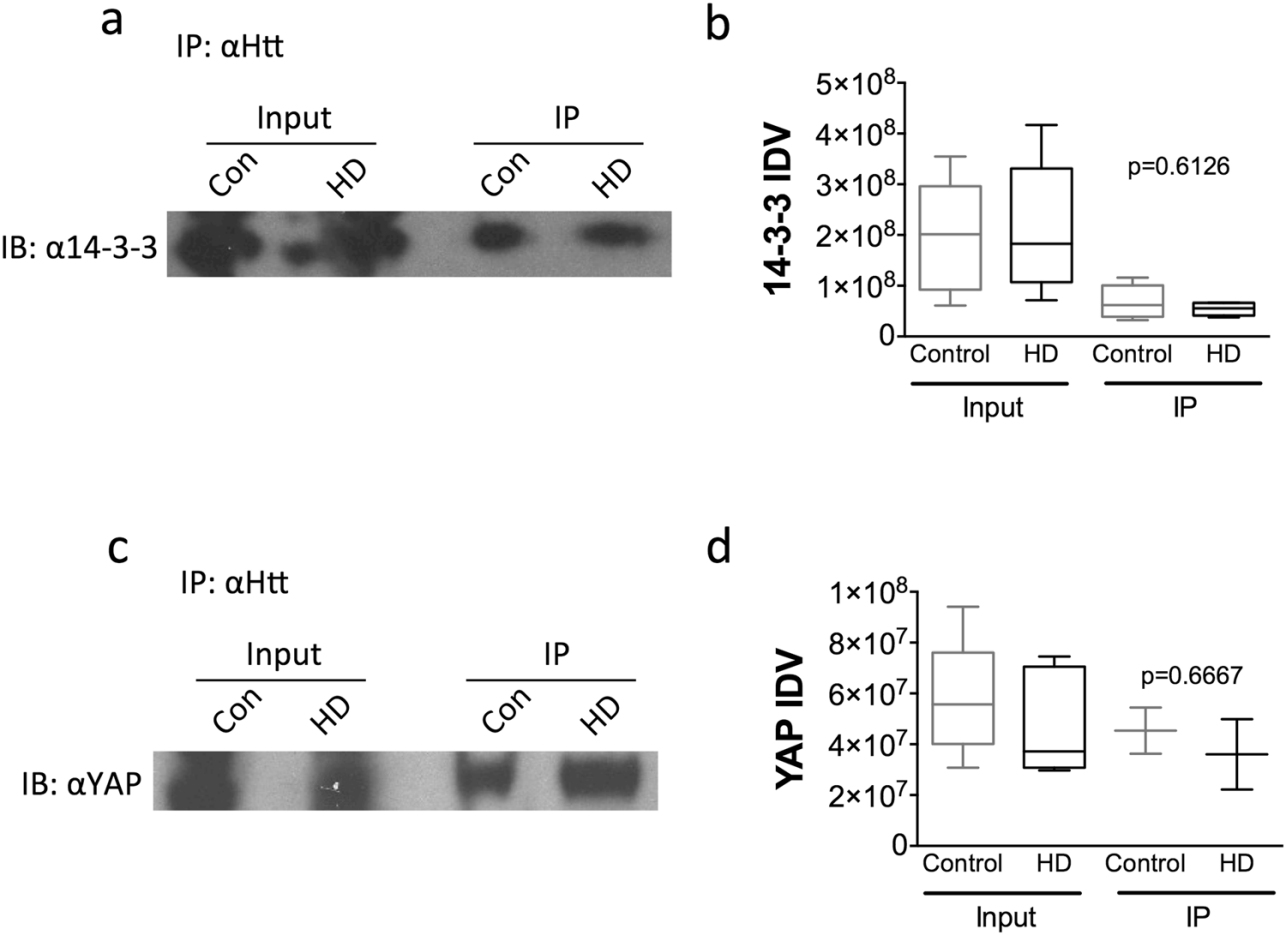
YAP-Htt-14-3-3 interactions. (**a**) Representative blot from a co-immunoprecipitation assay using human cortex with anti-14-3-3 antibody followed by immunoblotting with an anti-huntingtin (2166) antibody. (**b**) There was no change in 14-3-3 levels in the input samples (left) (p = 0.7209, Mann-Whitney *U* Test) or in 14-3-3/Htt interactions (right) in HD cortex (black; n = 8 input and IP) compared to control (grey; n = 8 input, n = 7 IP) as demonstrated in the IP samples (p = 0.6126, Mann-Whitney *U* Test). (**c**) Representative blot from a co-immunoprecipitation assay using human cortex with anti-YAP antibody followed by immunoblotting with an anti-huntingtin (2166) antibody. (**d**) There was no change in YAP levels in the input samples (left) (p = 0.1031, Mann-Whitney *U* Test) in HD cortex (black; n = 7) compared to control (grey; n = 7) or in YAP/Htt interactions (right) (p = 0.6667, Mann-Whitney U Test) in HD cortex (black; n = 7) compared to control (grey; n = 7) as demonstrated in the IP samples in HD cortex (black; n = 2) compared to control (grey; n = 2) (p = 0.6667, Mann-Whitney *U* Test). Data are presented as median and min-max values.

The chaperone 14-3-3, an abundant protein in the brain, is known to interact and sequester YAP in the cytoplasm^29,30^. It has also been shown to play a key role in the formation of Htt aggregates through its interaction with mHtt^31^. Given this interaction, we sought to determine if decreases in nuclear YAP are caused by increased interactions with 14-3-3 in the presence of mHtt. Co-immunoprecipitation (Co-IP) was performed using antibodies specific for YAP, 14-3-3, and Htt. The chaperone 14-3-3 was shown to interact with both wild-type Htt and mHtt (Fig. 4a,b) as previously shown by Shirasaki *et al*.^32^ but there was no difference between HD and control cortex (p = 0.6126, Mann-Whitney *U* Test). Moreover, YAP was also found to interact with wild-type Htt and mHtt (Fig. 4c,d) but there was no difference in interaction between HD and control cortex (p = 0.6667, Mann-Whitney *U* Test). Taken together, these findings indicate that while YAP, 14-3-3, and Htt may be in a complex together, there is no significant difference in this interaction in the presence of mHtt.

### Expression patterns of Hippo pathway components in human cortex

**Figure 5:**
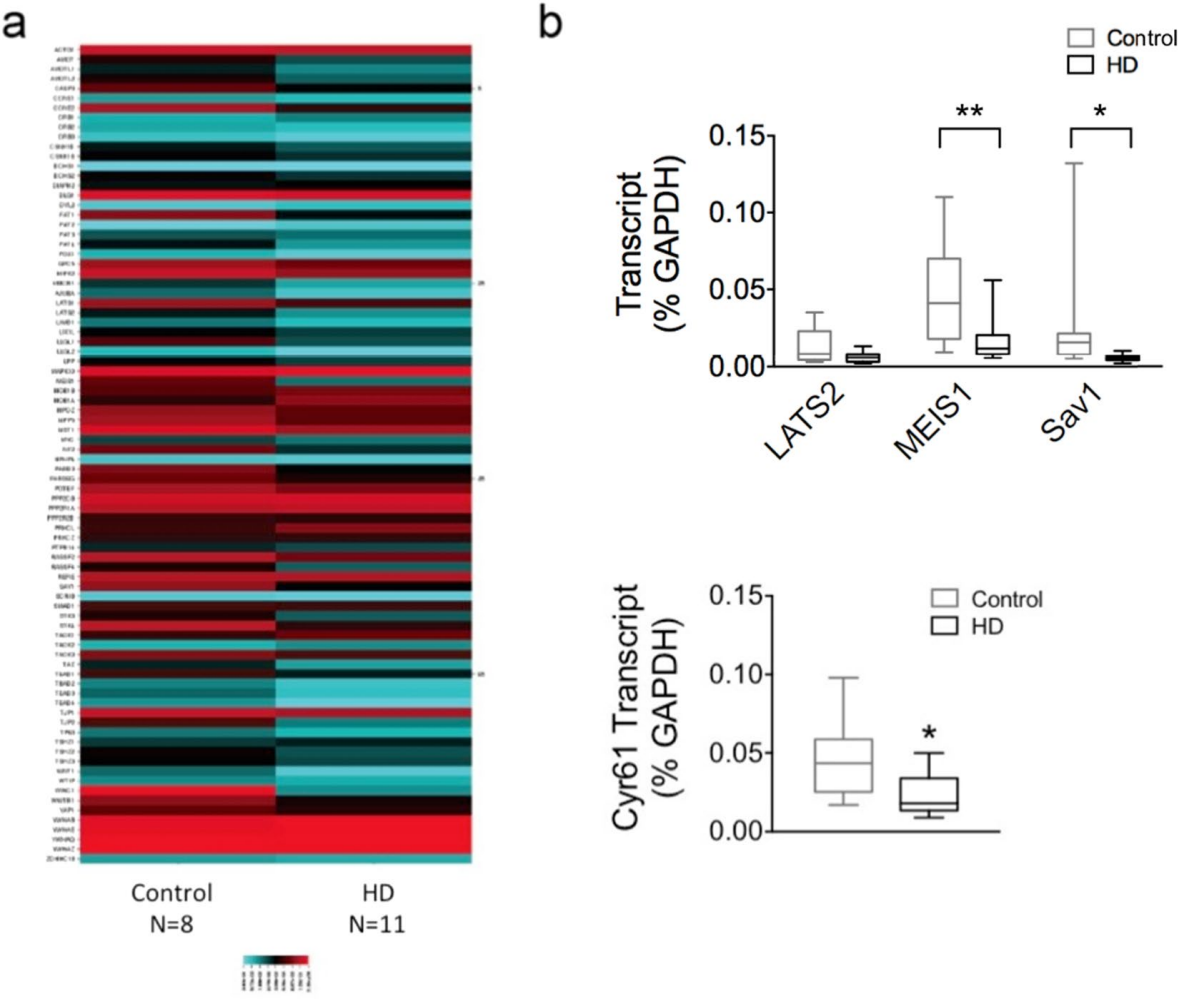
Expression patterns of Hippo pathway components in human cortex. (**a**) Heat map illustrating expression levels in Hippo pathway proteins from a gene expression array performed on control (n = 8) and HD (n = 14) human cortex samples. Colors indicate expression levels (blue = low to red = high). Overall there were more downregulated genes in human HD compared to control cortex. (**b**) Two-way ANOVA revealed a significant effect of genotype [F(2, 60) = 6.633, p = 0.0025] and treatment [F(1, 60) = 17.03, p = 0.0001] but no effect of interaction [F(2, 60) = 1.826, p = 0.1698] on gene expression from qPCR analysis. Sidak’s multiple comparison’s test demonstrated that there was a significant decrease in *Meis1* (p < 0.001) and *Sav1* (p < 0.05) expression in HD cortex compared to control but not a decrease in *Lats2* expression (p > 0.05). (**c**) qPCR findings also demonstrated a significant decrease in Cyr61, another known YAP target gene, expression in HD cortex (black; n = 13) compared to control (grey; n = 8) (p = 0.0132 Mann-Whitney *U* Test). Data are presented as medians and min to max. *p < 0.05, **p < 0.01.

In order to determine if the expression of the Hippo signaling pathway components and target genes are dysregulated in HD we used the RT^2^ Profiler PCR Array for Human Hippo Signaling Pathway and assessed alterations in transcript levels in post-mortem cortices from healthy controls (n = 8) and HD patients (n = 14). These arrays contain primers for 84 different Hippo pathway-related genes (Suppl. Table 1). The transcript level of each gene was normalized to the GAPDH mRNA level within each respective plate and expressed as 2^−ΔCT^. Two-way ANOVA demonstrated that there is a significant effect of genotype [F(1, 1258) = 5.189, p = 0.0229], gene [F(83, 1258) = 34.15, p < 0.0001], and genotype X gene interaction [F(83, 1258) = 2.421, p < 0.0001] revealing that the transcription of the Hippo pathway related genes is altered in HD postmortem cortex compared to controls, as illustrated by the heat map (Fig. 5a) and in Suppl. Table 1. To confirm some of the hits from the array findings, individualized RT-qPCR reactions were performed for three downregulated genes: *Lats2*, *Meis1*, and *Sav1* in the same HD tissue that was used for the Hippo array analysis. Two-way ANOVA revealed a significant effect of genotype [F(2, 60) = 6.633, p = 0.0025] and treatment [F(1, 60) = 17.03, p = 0.0001] but no effect of interaction [F(2, 60) = 1.826, p = 0.1698] on gene expression. Sidak’s multiple comparison’s test demonstrated that there was a significant decrease in *Meis1* (p < 0.01) and *Sav1* (p < 0.05) expression in HD cortex compared to control but no change in *Lats2* expression (p > 0.05) (Fig. 5b). We also measured the expression of a known YAP target gene, *cysteine-rich angiogenic inducer 61* (*Cyr61)*^18,20,33,34^, which was not represented on the array, and found a statistically significant decrease in its expression in HD cortex compared to control (p = 0.0132 Mann-Whitney *U* Test) (Fig. 5c). Together these findings demonstrate that Hippo signaling is altered in HD and suggest that the decrease in YAP nuclear activity is associated with subsequent transcriptional dysregulation which may be a significant contributor to neuronal injury in HD.

### YAP/TEAD interactions are decreased in HD

**Figure 6:**
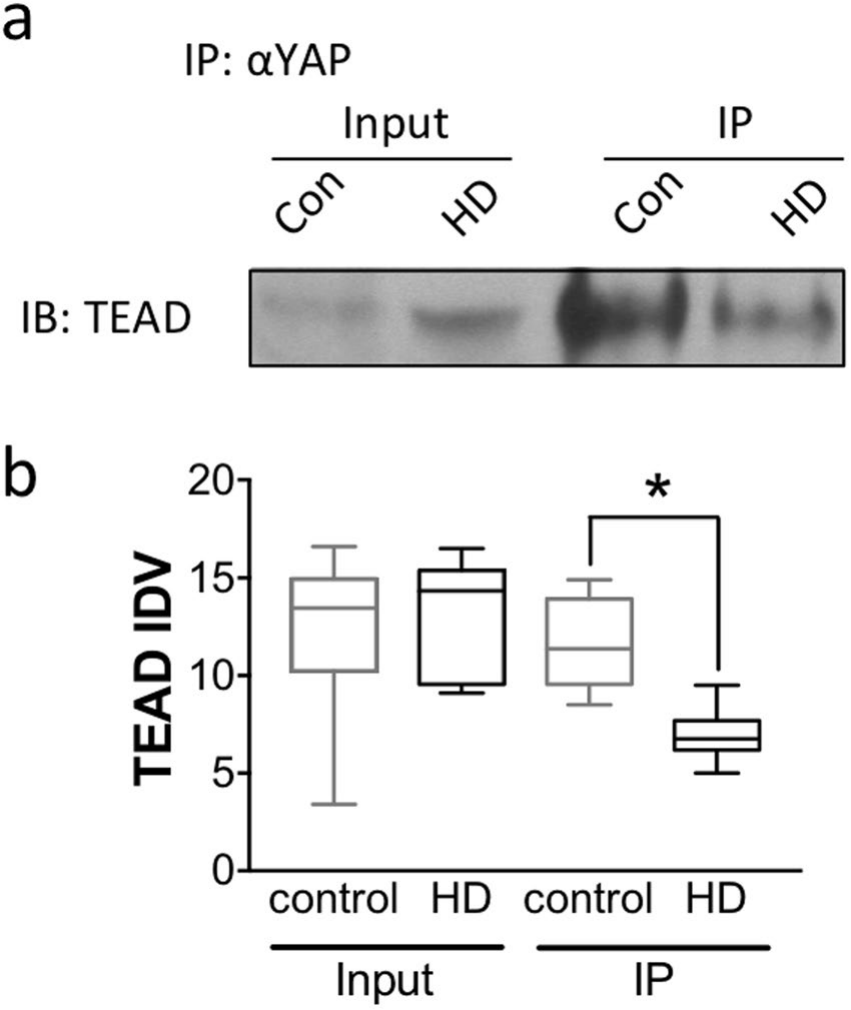
YAP/TEAD interactions are decreased in HD. (**a**) Representative blot from a co-immunoprecipitation assay using human cortex with anti-YAP antibody followed by immunoblotting with an anti-pan-TEAD antibody. (**b**) There was no change in TEAD levels in the input samples (p = 0.6857, Mann-Whitney *U* Test) (left) but there was a significant decrease in YAP/TEAD interactions (right) in HD cortex (black; n = 6) compared to control (grey; n = 7) as demonstrated in the IP samples (p = 0.0043, Mann-Whitney *U* Test). Data are presented as median and min-max values. *p < 0.05.

YAP overexpression induces the transcription of pro-survival genes^24,35^ by binding to TEAD and thereby controlling cell growth, proliferation and survival^36–38^. Given the alterations in Hippo pathway genes together with decreases in nuclear YAP levels we sought to examine whether YAP/TEAD interactions were altered in HD. Co-immunoprecipitation was performed using an anti-YAP antibody for immunoprecipitation (IP) and an anti-TEAD antibody for immunoblots in the same post-mortem HD and control cortical samples. The results demonstrate that while TEAD levels were not altered in input samples (p = 0.6857, Mann-Whitney *U* Test), there was a significant decrease in YAP/TEAD interactions in HD cortex compared to control as demonstrated in the IP samples (p = 0.0043, Mann-Whitney *U* Test) (Fig. 6a,b). These findings suggest that decreases in nuclear YAP leads to a decrease in YAP/TEAD interactions in HD that may translate to alterations in gene expression.

**Figure 7:**
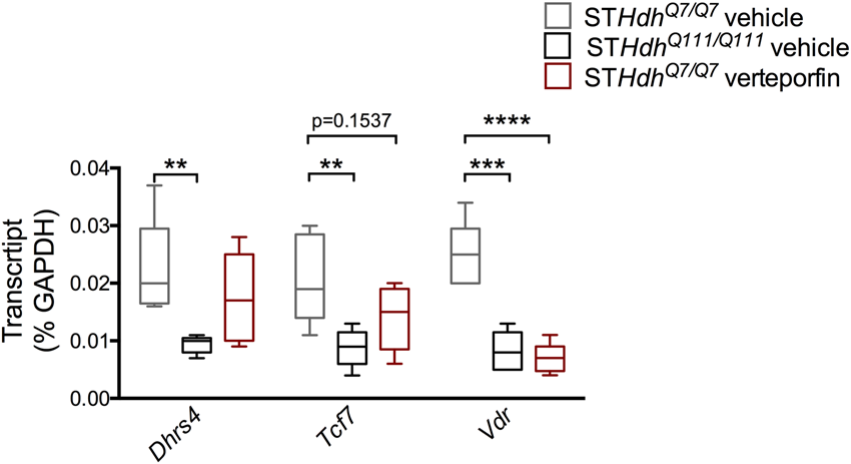
Disruption of YAP/TEAD interaction alters transcription. Two-way ANOVA demonstrated that there was a significant effect of gene [F(2, 12) = 23.87, p < 0.0001] but no effect of treatment [F(2, 36) = 1.144, p = 0.3298] or interaction [F(4, 36) = 2.019, p = 0.1124] on expression data from qPCR analysis. Tukey’s multiple comparison’s test a significant decrease in expression of the NADPH dehydrogenase (*Dhrs4*) (p = 0.0025), transcription factor 7 (*Tcf7*) (p = 0.0053), and vitamin D receptor (*Vdr*) (p = 0.0001) genes in untreated ST*Hdh*^*Q7/Q7*^ cells (light grey) compared to untreated ST*Hdh*^*Q111/Q111*^ cells (black; n = 5). In addition, verteporfin treatment (10 mM, 24 hours) in ST*Hdh*^*Q7/Q7*^ cells (red; n = 5) also significantly decreased *Vdr* expression (p < 0.0001, Tukey’s post-hoc test) and produced a trend towards a decrease in *Tcf7* expression (p = 0.1537, Tukey’s post-hoc test) compared to untreated ST*Hdh*^*Q7/Q7*^ cells. However, verteporfin treatment did not affect *Dhrs4* (p = 0.3535, Tukey’s post-hoc test) expression in ST*Hdh*^*Q7/Q7*^ cells. Data are presented as median and min-max values. **p < 0.01, **p < 0.001, ****p < 0.0001.

Next we sought to determine if transcriptional alterations due to the presence of mHtt could be recapitulated by disrupting YAP/TEAD interactions. Wild-type ST*Hdh*^*Q7/Q7*^ cells, immortalized striatal cell lines derived from the Htt CAG knock-in mice^39^, were treated with 10 μM verteporfin, a compound that disrupts YAP/TEAD interactions^40^, for 24 hours and alterations in gene expression were measured. A two-way ANOVA demonstrated that there was a significant effect of gene [F(2, 36) = 23.87, p < 0.0001] but no effect of treatment [F(2, 36) = 1.144, p = 0.3298] or interaction [F(4, 36) = 2.019, p = 0.1124] on gene expression data. Tukey’s multiple comparison’s test demonstrated that there was a significant decrease in the expression of NADPH dehydrogenase (*Dhrs4*) (p = 0.0025), transcription factor 7 (*Tcf7*) (p = 0.0053), and vitamin D receptor (*Vdr*) genes (p = 0.0001) in untreated ST*Hdh*^*Q7/Q7*^ cells compared to untreated ST*Hdh*^*Q111/Q111*^ cells, in agreement with previous findings^41,42^. Importantly, verteporfin treatment in ST*Hdh*^*Q7/Q7*^ cells also significantly decreased *Vdr* expression (p < 0.0001, Tukey’s post-hoc test) and resulted in a trend towards a decrease in *Tcf7* expression (p = 0.1537, Tukey’s test) compared to untreated ST*Hdh*^*Q7/Q7*^ cells. However, verteporfin treatment did not affect *Dhrs4* (p = 0.3535, Tukey’s post-hoc test) expression in ST*Hdh*^*Q7/Q7*^ cells (Fig. 7). These results demonstrate that disruption of YAP/TEAD interactions recapitulates decreases in *Vdr* expression induced by the presence of mHtt.

## Discussion

Alterations in the Hippo signaling pathway are traditionally linked to organ size and cancer. However, here we demonstrate that YAP nuclear activity is also altered in HD and may be linked to its pathogenesis as suggested by previous studies^25,26,28^. The current study demonstrates that there is a significant decrease in nuclear YAP levels in HD cortex as well as in HD ES-derived neuronal stem cells (Fig. 1). In addition, there is a concomitant decrease in YAP/TEAD interactions and the expression of Hippo pathway related genes is altered in HD patient cortex (Figs 5 and 6). Our results also suggest that decreases in nuclear YAP, YAP/TEAD interaction, and transcriptional dysregulation in HD, may be due to an increase in pMST1 (active form) levels and not due to an increase in the interaction between YAP and the chaperone 14-3-3 in the presence of mHtt (Fig. 4a,b) or LATS activation in HD (Fig. 3e,f), as suggested previously^26,28^. Lastly, increases in MST1 activation and decreases in nuclear YAP levels were recapitulated in the homozygous Hdh^*Q111/Q111*^ knock-in mice (Fig. 3g–k), confirming previously published results^26^.

YAP levels were assessed in both ES- and iPSC-derived control and HD NSCs. While total YAP levels were not altered between HD and control cell lines, there was a significant decrease in nuclear YAP levels in the ES-derived HD NSCs only. Although nuclear YAP levels were not statistically different between the control and HD iPSC-derived NSCs (p = 0.0931) there was a trend towards a decrease in nuclear YAP levels between HD4 and WT97 cells (Fig. S4). This subtle discrepancy in nuclear YAP levels between the two NSC lines may be due to the CAG repeat length between the HD ES-derived (46 CAG repeats) and HD iPSC-derived (72 CAG repeats) NSCs or differences in the cells of origin. It should be noted that the CAG repeat length of the ES-derived NSCs is similar to adult HD and recapitulates the significant decrease in nuclear YAP levels measured in post-mortem brain samples.

Decreases in YAP nuclear activity are linked to a decrease in cell survival^19,22^. In HD, decreases in YAP were previously linked to transcriptional repression-induced atypical cell death (TRIAD)^25^ as well as mHtt-induced neuronal death termed ‘ballooning cell death’ (BCD)^26^. Specifically, YAP and YAPΔC, a neuronal isoform of YAP with a truncation of the C-terminus, were shown to inhibit TRIAD in HD^25^ suggesting that YAP and YAPΔC are neuroprotective^25^. In a more recent follow up study, Mao and colleagues demonstrated that the expression of YAP and YAPΔC decreased mHtt-induced BCD, similar to TRIAD, in primary neuronal cultures^26^. Moreover, their findings demonstrated that BCD was exacerbated by increases in phosphorylated YAP (inactive and cytoplasmic) and the concomitant decrease in YAP/TEAD mediated transcription. Their findings suggested a role for a p73 regulated mechanism and, similar to the previous study, suggested a protective role for YAPΔCs in HD^26^. Although our study confirmed decreases in neuronal and nuclear YAP together with decreases in YAP-TEAD interactions as previously reported, we were unable to replicate the decrease in YAPΔC isoform (Fig. 2b) in HD cortex^25,26,28^ and therefore we did not assess the role of p73 in this study. While our findings may differ from the previously described results, this discrepancy may be explained by a number of factors including the quality of post-mortem tissue, differences in antibodies used, as well as subtle differences in methodology. However, additional studies are required in order to validate or refute this finding. In addition, Mao and colleagues demonstrated that YAP co-localized in Htt aggregates in the R6/2 and CAG knock-in Hdh^*Q111/Q111*^ mice^26^. Although we were not able to replicate these exact findings, we were able to demonstrate that YAP and Htt interact by co-IP (Fig. 4c,d). However, there was no significant difference in Htt-YAP interactions between control and HD cortical samples (Fig. 4c,d), suggesting that although YAP may be sequestered in Htt aggregates as previously suggested, Htt-YAP interactions are not altered by the presence of mHtt.

Although increases in MST1/2 have been implicated in other neurodegenerative diseases and cause neuronal death^27,43–46^ the pathogenic role of MST1/2 has not been elucidated in HD. Our findings demonstrate a trend towards an increase in phosphorylated MST1/2 (pMST1/2), the activated kinase, in the cortex of HD patients (Fig. 3a,b) as well as a significant increase in the cortex, but not in the striatum, of the homozygous Hdh^*Q111/Q111*^ knock-in mouse (Fig. 3g–j). Together, these findings suggest that increases in pMST1/2 may reduce YAP’s nuclear activity thereby causing neuronal death in HD. Our findings also demonstrate an increase in pYAP in both HD human and mouse brain (Fig. 3a,b,g,h) suggesting that increases in YAP phosphorylation lead to decreases in nuclear YAP levels. LATS1/2 is the kinase responsible for directly phosphorylating YAP in the Hippo pathway^35,38^. More recently, Yamanishi and colleagues demonstrated that LATS1 activation is increased and is linked to structural changes in ER and necrosis in human HD cortical neurons, as well as in the striatum of Hdh^*Q111/Q111*^ mice^28^. Here, we also measured alterations in LATS2 and pLATS2 (active form) in post-mortem cortex from HD patients and control. However, we were unable to replicate the previous findings demonstrating increases in LATS1 in HD, as there was no significant difference in LATS2 or pLATS2 levels in HD in our study (Fig. 3e,f). This may be due to the fact that the function of LATS1/2 in inhibiting YAP is cell type-dependent^37^. In addition, the involvement of other kinase proteins such as Akt, which has been previously reported to phosphorylate YAP at serine 127^47^, cannot be ruled out. Activation of LATS has also been linked to Plk1, the apoptosis promoter kinase, which shifts YAP transcriptional partners from TEAD to p73 thereby altering the signal from pro-survival to a pro-apoptotic pathway^28^. Given that we did not measure any significant change in LATS in HD human tissue we did not assess alterations in Plk1 or p73 in this study. Future studies will assess this potential mechanism in our HD samples.

YAP phosphorylation on S127 is essential for its interaction with the chaperone 14-3-3^35,38^ leading to cytoplasmic retention and the loss of nuclear YAP^38,48,49^. 14-3-3 proteins are abundant molecules in the brain with the capacity to affect the localization and activity of proteins^50^. In HD, the Htt-containing interactome was shown to be highly enriched with proteins involved in 14-3-3 signaling^32^. In addition, it has been suggested that 14-3-3 *ζ* can scavenge misfolded Htt and facilitate the formation of aggregates^31,51^ indicating a role for 14-3-3 in Htt aggregate formation. Given that 14-3-3 interacts with both YAP and Htt, we assessed if this interaction was altered in HD. Our results indicate that while both Htt and YAP interact with 14-3-3, there is no significant difference in this interaction in the presence of mHtt (Fig. 4). Together these results suggest that decreases in nuclear YAP in HD are not due to an increase in interaction with 14-3-3. Lastly, changes in nuclear YAP levels may be due to alterations in nucleocytoplasmic transport as suggested by recent studies demonstrating defects in the transport of proteins and mRNA in HD^52,53^. Intranuclear Htt aggregates were shown to sequester Gle1 and RanGAP1, both major regulators of nucleocytoplasmic transport in HD^52^. However, future studies will be needed to assess this hypothesis.

A key mechanism underlying HD pathogenesis is transcriptional dysregulation (reviewed in^3,54^). Aberrant transcriptional alterations occur early in HD and have been demonstrated in patients as well as in multiple cellular and animal models of HD^3^. YAP is a transcriptional coactivator which, upon translocation into the nucleus, is able to regulate gene expression through its interaction with TEAD1–4^38^. Previous studies have demonstrated that knockdown of TEADs or disruption of the YAP/TEAD interaction decreases the expression of the majority of YAP target genes^55^. Our study confirms these results and previous findings in HD^26^, demonstrating a decrease in YAP/TEAD interactions as well as a decrease in the expression of several Hippo target genes (Figs 5 and 6). In addition, our findings demonstrate that YAP/TEAD interactions are necessary for normal gene expression as treatment with verteporfin, a compound that disrupts YAP/TEAD interaction^56^, decreased *Vdr* expression and produced a trend towards a decrease in *Tcf7* expression in the wild-type cells (Fig. 7). Although more YAP/TEAD target genes will need to be assessed in future studies, these results demonstrate that disruption of YAP/TEAD interactions may recapitulate some of the transcriptional changes induced by the presence of mHtt.

Two previously published studies have indicated that the incidence of cancer is lower in HD patients than in controls^57,58^. Given the decreases in neuronal as well as nuclear YAP levels in HD along with YAP’s role in tumorigenesis, this is not a surprising finding as the major downstream effector of the Hippo pathway, YAP functions as an oncogene^59–61^. Furthermore, increases in YAP expression and nuclear localization are consistently observed in multiple types of cancers^35,38,62^. In HD, there is a significant decrease in neuronal nuclear YAP levels as demonstrated here and as reported previously^25,26,28^, the opposite scenario than what happens in cancer. Given these findings, recent studies have focused on elucidating the role of Htt in cancer^63^ suggesting that interactions of wild-type or mHtt with the Hippo pathway may be critical in cancer pathogenesis as well as in HD.

Our study demonstrates that pMST1/2 and pYAP levels are increased in post-mortem samples from HD patients (Fig. 3a,b) as well as in the cortex and striatum of 10-month old homozygous Hdh^*Q111/Q111*^ mice (Fig. 3g–j). Additionally, there is a concomitant decrease in nuclear YAP in HD (Fig. 1) suggesting that activation of MST1/2 upstream of YAP may lead to alterations in YAP levels and nuclear localization and thereby provide a new target for therapy. Indeed, the study by Mao and colleagues demonstrated a decrease in nuclear YAP levels in homozygous CAG knock-in Hdh^*Q111/Q111*^ mice and that treatment with sphingosine-1-phosphate (S1P), an inhibitor of the Hippo pathway, reversed motor deficits and improved YAP expression in the homozygous Hdh^*Q111/Q111*^ mice^26^. Our findings of elevated pMST1/2 together with these previously published results suggest that inhibiting MST1/2, thereby increasing YAP activity, may confer neuroprotection in HD, as previously described in a mouse model of amyotrophic lateral sclerosis^64^. Thus, the use of compounds that target the Hippo pathway such as S1P or other ligands of GPCR receptors upstream of the Hippo pathway should be considered for the treatment of HD. Alternatively, the use of genetic approaches such as knockdown of MST1/2 using shRNA to decrease YAP phosphorylation and enhance its nuclear localization, may provide another avenue for therapeutic development in HD.

## Methods

All methods were carried out in accordance with the guidelines and regulations of Massachusetts General Hospital and approved by the Massachusetts General Hospital licensing committees.

### Human samples

Post-mortem cortical tissue from control and Huntington’s disease patient brains were provided by the Massachusetts Alzheimer’s Disease Research Center (ADRC) with approval from the Massachusetts General Hospital IRB (1999p009556). The PMI for post-mortem tissue ranged between 6–20. We used frontal cortex, Brodmann area 9 (BA9) regions for the outlined studies. The HD Vonsattel Grades ranged between Grade 2 (n = 5) and Grade 3 (n = 7) with an equal number of males and females distributed between the groups. The average age at death was 61.8 ± 13.3 years. The use of discarded tissue does not meet the Massachusetts General Hospital IRB definition of human subjects research and therefore did not require obtaining informed consent.

### Animals

All animal work was performed according to the guidelines set by the Massachusetts General Hospital Subcommittee on Research Animal Care. The cortex and striatum of the CAG knock-in Hdh^*Q7/Q7*^ and Hdh^*Q111/Q111*^ mouse model was a generous gift from Drs. Lianna Orlando and Kim Kegel (Massachusetts General Hospital). Hdh mice contain chimeric human and mouse *HD/Hdh* exon 1 with a sequence encoding for the human polyproline tract together with either a full-length version of either wild-type (containing 7 glutamines) or mtt (111 glutamines) Htt. We used both the cortex and striatum from the wild-type (Hdh^*Q7/Q7*^) and homozygous (Hdh^*Q111/Q111*^) genotypes^39,65^.

### Cell Culture

#### HD neuronal stem cells

Human embryonic stem cells (hESCs) were obtained from WiCell (WA09, unaffected control) and GENEA (GENEA018, HD with 17/46 CAGs) and maintained on MEF feeders prepared using mitomycin C. hESCs were grown in normal growth medium (Knockout DMEM/F12 (Life Technologies) with 20% knockout serum replacement (Life Technologies), 1 × MEM non-essential amino acids (Life Technologies), 1 mM L-glutamine (Life Technologies), 5 mL penicillin/streptomycin (Life Technologies), and 100 µM 2-mercaptoethanol). The GENEA018 hESCs were grown on collagen in Genea M2 medium (from GENEA).

Human induced pluripotent stem cells (iPSCs) were acquired from NINDS Repository at the Coriell Institute for Medical Research (line #ND36997, 33 CAG -unaffected control) and from NIGMS Repository (HD-iPS4, HD with 17/72CAG) from Dr. George Daley of the Children’s Hospital Boston Stem Cell Program.

Human neuronal stem cells (NSCs) were differentiated based on a previously established protocol described for mouse ES cells^66^. Briefly, stem cells were passaged in DMEM (Life Technologies) with 1 mg/mL collagenase type I (Life Technologies) on to 0.1% gelatin coated plates (Sigma) containing NSC media (DMEM/F12 (Life Technologies) with 2 mM L-glutamine, retinoic acid-free B27 supplement (Life Technologies), 5 mL penicillin/streptomycin (Life Technologies), and N2 supplement (Invitrogen, Carlsbad, CA)). The media was also supplemented with 100 ng/mL EGF (Invitrogen), 100 ng/mL FGF (Invitrogen), 5 µg/mL heparin (Sigma), and 1% knockout serum replacement. Once a 60–80% confluent monolayer of bipolar cells formed, cells were passaged with Accutase (Sigma), and pelleted by centrifugation for 3 minutes at 1200 rpm. Cells were then re-plated as a single cell suspension onto 0.1% gelatin coated plastic, in NSC media lacking additional knockout serum and supplemented with 20 ng/mL FGF, 20 ng/mL EGF, and 5 µg/mL heparin. The cells were routinely tested for expression of the radial glial marker Nestin (Millipore, Burlington, MA) by immunofluorescence and for normal and mutant huntingtin expression using anti-Htt antibody^67^ by Western blot.

#### STHdh cells

Striatal cell lines were established from wild-type (Hdh^*Q7/Q7*^) and Hdh (Hdh^*Q111/Q111*^) knock-in embryonic mice^39^ and were a generous gift from Dr. Marcy MacDonald (Massachusetts General Hospital). ST*Hdh* cell lines express full-length versions of either wild-type (containing 7 glutamines) or mutant (111 glutamines) Htt. Two different cell lines were used, corresponding to wild-type (ST*Hdh*^*Q7/Q7*^), and homozygous (ST*Hdh*^*Q111/Q111*^) genotypes. ST*Hdh* cells were used in passages 5 to 14. Cells were kept in Dulbecco’s modified Eagle’s medium (DMEM) (Life Technologies, Carlsbad, CA) plus 10% fetal bovine serum and 1% of the antibiotics penicillin streptomycin and geneticin at 33 °C for propagation, and were placed at 39 °C for 48 h to stop their division.

#### Verteporfin Treatment

Verteporfin (Sigma, St. Louis, MO) was made into a 1 mM stock solution using sterile, filtered DMSO and diluted into DMEM for treatment. ST*Hdh* cell lines were treated with 10 µl of either vehicle (DMEM with 100 μl DMSO) or 10 μM verteporfin (DMEM with 100 μl 1 mM verteporfin) for 24 hr. The cells were washed with PBS and pelleted for further use.

#### Western blotting

Western blots were carried out as previously described^42,68^. Briefly, 75 µg of protein was resuspended in sample buffer, boiled at 95 °C for 5 min, and fractionated on a 4–20% glycine gels (Invitrogen) for 90 min at 120V. Proteins were transferred to a PVDF membrane in an iBlot Dry Blotting System (Invitrogen), and the membrane was then blocked with 5% milk in tris-buffered saline with Tween 20 (TBST) before immunodetection with antibodies specific for the following: YAP (Cell Signaling Technology Danvers, MA), pYAP (Cell Signaling Technology), MST1 (Cell Signaling Technology), pMST1/2 (Cell Signaling Technology), LATS2 (Bethyl Laboratories Montgomery, TX), pLATS2 (Abnova Taipei, Taiwan) and GAPDH (Millipore). Primary antibody incubation overnight was followed by 4 washes (10 min, RT) in TBST before incubation with the secondary antibody for 1 h (HRP-conjugated goat anti-rabbit IgG Jackson ImmunoResearch Laboratories, West Grove, PA; and HRP-conjugated goat anti-mouse Bio-Rad Laboratories Hercules, CA). After 4 washes in TBST (10 min, RT) proteins were visualized using the ECL detection system (NEN, Boston, MA). Coomassie gels were used to ensure equal protein loading for western blots.

#### Immunostaining and Image Analysis

Fresh frozen brain tissue was sectioned at 10 µm and stained using previously described methods^20^. In short, tissue sections were fixed with 4% paraformaldehyde and blocked in a mixture of phosphate-buffered saline (PBS), 5% BSA, and normal goat serum. Co-staining was done using antibodies specific for YAP (Novus Biologicals, Littleton, CO), GFAP (Cell Signaling), and NeuN (Millipore) overnight at 4 °C. Following three washes with PBS, sections were incubated with Cy3-conjugated goat anti-mouse and FITC-conjugated goat anti-rabbit antibodies (Jackson ImmunoResearch, West Grove, PA). The data was analyzed using open source software from the National Institutes of Health (ImageJ). Three 40× fields were photographed from each section of HD and control tissue. For each field, the following was counted: (1) the total number of nuclei staining positive for DAPI; (2) the number of NeuN-positive cells; and (3) the number of GFAP-positive cells. The number of nuclei staining positive for YAP against background were then counted manually and expressed as a percentage of the total nuclei. The same was done for YAP in NeuN-positive and GFAP-positive cells.

#### Nuclear/Cytoplasmic Fractionation

Nuclear and cytoplasmic fractions were extracted from human post-mortem cortex using the CelLytic NuCLEAR Extraction Kit (Sigma). Briefly, tissue was homogenized in hypotonic lysis buffer (10× lysis buffer, dH2O, 0.1M DTT, and protease inhibitor), centrifuged for 5 min at 420 × g. The supernatant was discarded and the pellet was re-suspended in lysis buffer and sonicated. Following sonication, the samples were centrifuged for 20 min at 10,000 × g and the supernatant was retained as the cytoplasmic fraction. The remaining pellet was re-suspended in extraction buffer, sonicated, and agitated at 4 °C for 30 min. The sample was centrifuged for 5 min at 20,000 × g and the supernatant was collected as the nuclear fraction.

#### Co-immunoprecipitation

The co-immunoprecipitations studies were carried out as previously described^69^. Briefly, using post-mortem human cortex homogenates, 150 μg of protein was incubated with either 5 μl anti-YAP antibody (Novus Biologicals), anti-14-3-3- antibody (ThermoFisher Scientific Waltham, MA), or anti-Htt MAB2166 antibody (Millipore) and GAL4 immunoprecipitation buffer (2.5 ml 5M NaCl, 500 μl EDTA, 500 μl P-40, 0.3 g Tris Base, and dH2O to 50 ml). After incubation at 4 °C for 3.5 hours, magnetic protein A beads (Invitrogen) were added (20 μl per sample) and then left spinning overnight at 4 °C. Samples were placed on a magnetic rack to facilitate removal of the supernatant, and washed with GAL4. Samples were then boiled with 20 μl of sample buffer for 5 min at 95°.

For the input samples, 75 μg of protein was resuspended in sample buffer and boiled at 95 °C for 5 min. The samples were then loaded into a 4–20% glycine gel and the protocol for a Western blot was followed. The following were used to immunoblot: pan-TEAD (Cell Signaling Technology), 14-3-3 (ThermoFisher Scientific), Huntingtin (Millipore), pYAP (Cell Signaling Technology).

#### RNA extraction and reverse transcription

Cortical tissue and ST*Hdh* cell lines were homogenized in TRI Reagent (Sigma) and total RNA was extracted from cell lines using an RNeasy kit (Qiagen, Valencia, CA) according to manufacturer’s instructions and as previously described^68,70^. Reverse transcription reactions were performed using iScript Reverse Transcription Supermix for RT-qPCR (Bio-Rad, Hercules, CA) in an iCycler (Bio-Rad) (25 °C for 10 min, 42 °C for 50 min, 70 °C for 15 min).

#### Hippo Pathway Arrays and Quantitative Real-Time PCR

Assessment of gene expression was done using the RT^2^ Profiler PCR Array for Human Hippo Signaling Pathway, with SYBR-green PCR Master Mix (Qiagen) which contains primer sets for 84 different Hippo pathway-related genes. Transcript levels are expressed as 2^−ΔCT^, where the ΔCT is the difference between the cycle thresholds of the gene of interest and the GAPDH levels within each array for each sample. Confirmatory qPCR was subsequently performed with the following primers: *Sav1* forward: 5′-GTGCTCCTAGTGTACCTCGGT-3′ and reverse: 5′-CTCGTGCGTAAACCTGAAGC-3′; *Lats2* forward: 5′-TCATCCACCGAGACATCAAGCC-3′ and reverse: 5′-TTGTGAGTCCACCTGAACCCATGG-3′; *Meis1* forward: 5′-ATGTGACAATTTCTGCCACCG-3′ and reverse: 5′-CCTGAACGAGTAGATGCCGTG-3′, genes that were shown to be downregulated from the arrays, and normalized to *Gapdh* (forward: 5′-CACCAGGGCTGCTTTTAACTC-3′ and reverse: 5′-TGGAAGATGGTGATGGGATTT-3′) in an iCycler (95 °C for 10 min, 95 °C for 15 sec, and 59 °C for 1 min for a total of 40 cycles). In addition, supplementary qPCR was also performed for cysteine-rich angiogenic protein 61 (*Cyr61*), another YAP target gene that was not found on the array with the following primers: forward: 5′-TTCTTTCACAAGGCGGCACTC-3′ and reverse: 5′-AGCCTCGCATCCTATACAACC-3′. Threshold amplification cycle numbers (Ct) were measured using an iCycler thermal cycler (Bio-Rad), with continuous SYBR Green monitoring.

Following verteporfin treatment the following primers were used to assess gene expression in ST*Hdh* cells: *Vdr* forward: 5′-TGAAGAGGAAGGAGGAAGAGG-3′ and reverse: 5′-GGGTCGTAGGTCTTGTGGTG-3′; *Dhrs4* forward: 5′-GTAAGAAAGTGCTCATCGTC-3′ and reverse: 5′-ATCATTTCTTGTGGCCCTTG-3′; and *Tcf7* forward: 5′-TGTGAACTCCTTGCTTCTGG-3′ and reverse: 5′-CCCAGCTTTCTCCACTCTAC-3′.

#### Statistics

Normal distributions of data were not assumed regardless of sample size or variance. Box plots are used for graphical representation with the central line representing the median, the edges representing the interquartile ranges, and the whiskers representing the minimum and maximum values, respectively. Sample sizes are included in the figure legends for clarity purpose. Comparisons for unrelated samples were performed using a Mann-Whitney *U* test followed by Dunn’s multiple comparison post-tests or a two-way ANOVA followed by Sidak’s or Tukey’s multiple comparisons post-tests at a significance level (α) of 0.05. Exact P values (two-tailed) are reported.

## Electronic supplementary material

Supplementary Information
