# Supplementary material for: Hippo Signaling Pathway Dysregulation in Human Huntington’s Disease Brain and Neuronal Stem Cells
Source: Sci Rep. 2018 Jul 27;8:11355. doi: 10.1038/s41598-018-29319-4 (PMC6063913; doi:10.1038/s41598-018-29319-4)
Supplement: Supplementary file 1 — Supplementary Information [file 41598_2018_29319_MOESM1_ESM.pdf]

Hippo Signaling Pathway Dysregulation in Human Huntington's Disease Brain and Neuronal Stem Cells

Kaly A. Mueller[1]*, Kelly E. Glajch[1]*, Megan N. Huizenga[1], Remi A. Wilson[1], Eric J. Granucci[1], Amanda M. Dios[1], Adelaide R. Tousley[2], Maria Iuliano[2], Elizabeth Weisman[2], Michael J. LaQuaglia[3], Marian DiFiglia[2], Kimberly Kegel-Gleason[2], Khashayar Vakili[3], Ghazaleh Sadri-Vakili[1]

*These authors contributed equally

[1]NeuroEpigenetics Laboratory, MassGeneral Institute for Neurodegenerative Disease (MIND), Massachusetts General Hospital, Boston, MA, 02129-4404, USA

[2]Cellular Neurobiology Laboratory, MassGeneral Institute for Neurodegenerative Disease (MIND), Massachusetts General Hospital, Boston, MA, 02129-4404, USA

[3] Department of Surgery, Boston Children's Hospital

Corresponding author:

Ghazaleh Sadri-Vakili, Ph.D., MassGeneral Institute for Neurodegenerative Disease, Massachusetts General Hospital, Bldg 114 16th Street, R2200, Charlestown, MA 02129

Telephone: 617-724-1487

Fax: 617-724-1480

Email: gsadrivakili@mgh.harvard.edu

Keywords: Huntington's disease, transcription, YAP, Hippo pathway, MST1

Running Title: Decreased YAP In HD

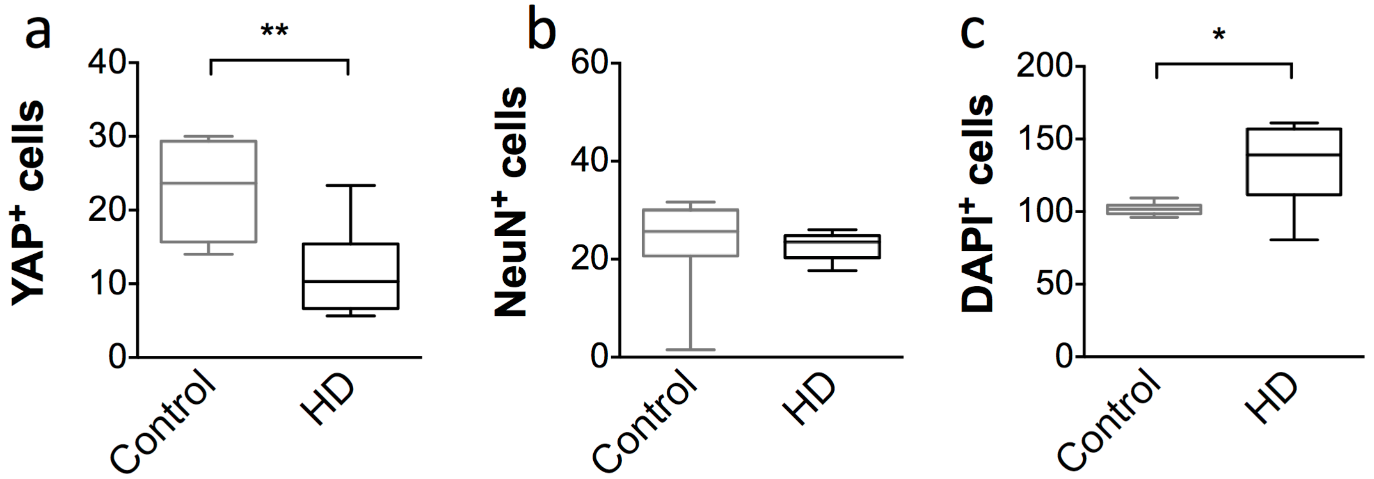

a
**
YAP+ cells
40
30
20
10
0
Control
HD
b
NeuN+ cells
60
40
20
0
Control
HD
c
*
DAPI+ cells
200
150
100
50
0
Control
HD

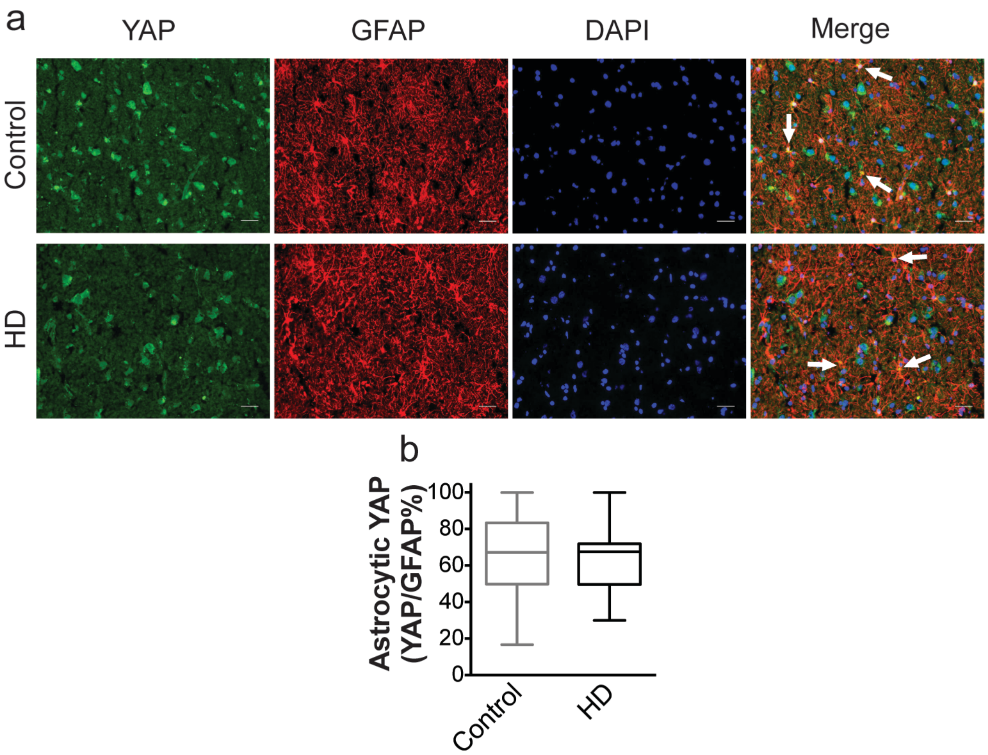
a
YAP
GFAP
DAPI
Merge
Control
HD
b
Astrocytic YAP
(YAP/GFAP%)
100
80
60
40
20
0
Control
HD

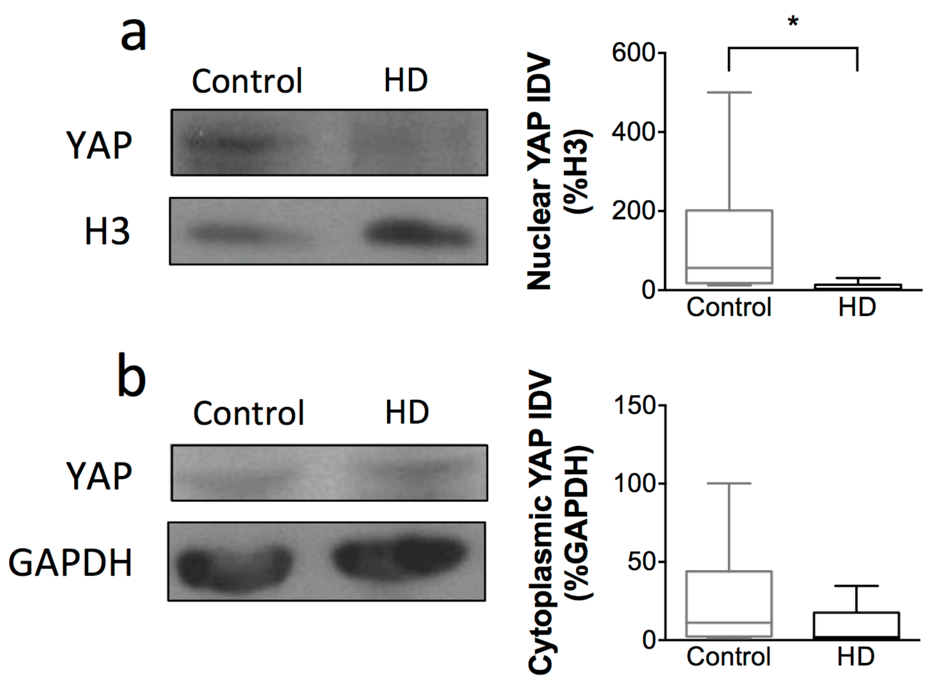

a
Control
HD
YAP
H3
Nuclear YAP IDV (%H3)
600
400
200
0
*
Control
HD
b
Control
HD
YAP
GAPDH
Cytoplasmic YAP IDV (%GAPDH)
150
100
50
0
Control
HD

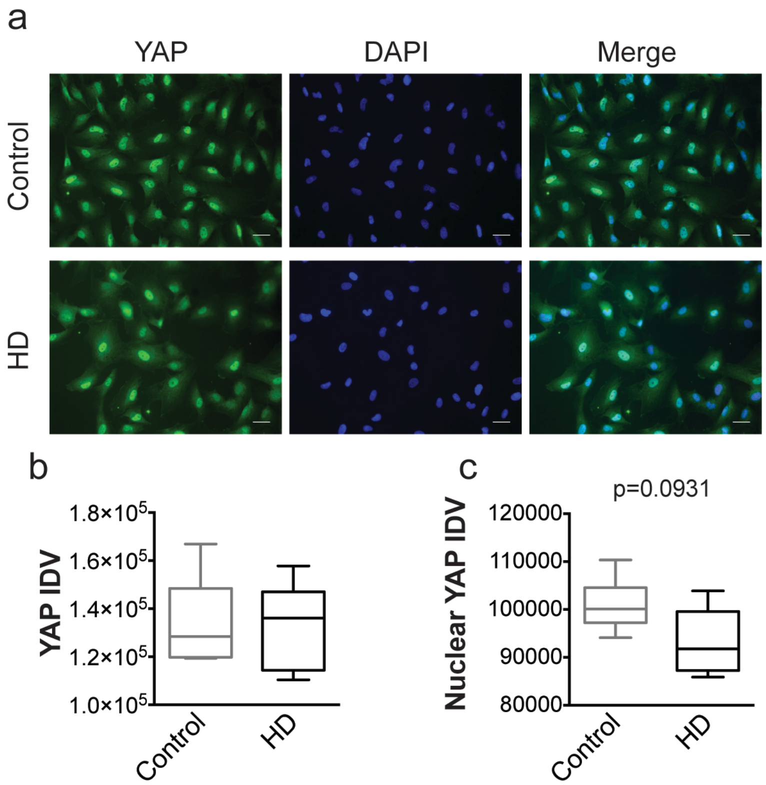

a
YAP
DAPI
Merge
Control
HD
b
YAP IDV
1.8×10^5
1.6×10^5
1.4×10^5
1.2×10^5
1.0×10^5
Control
HD
c
p=0.0931
Nuclear YAP IDV
120000
110000
100000
90000
80000
Control
HD

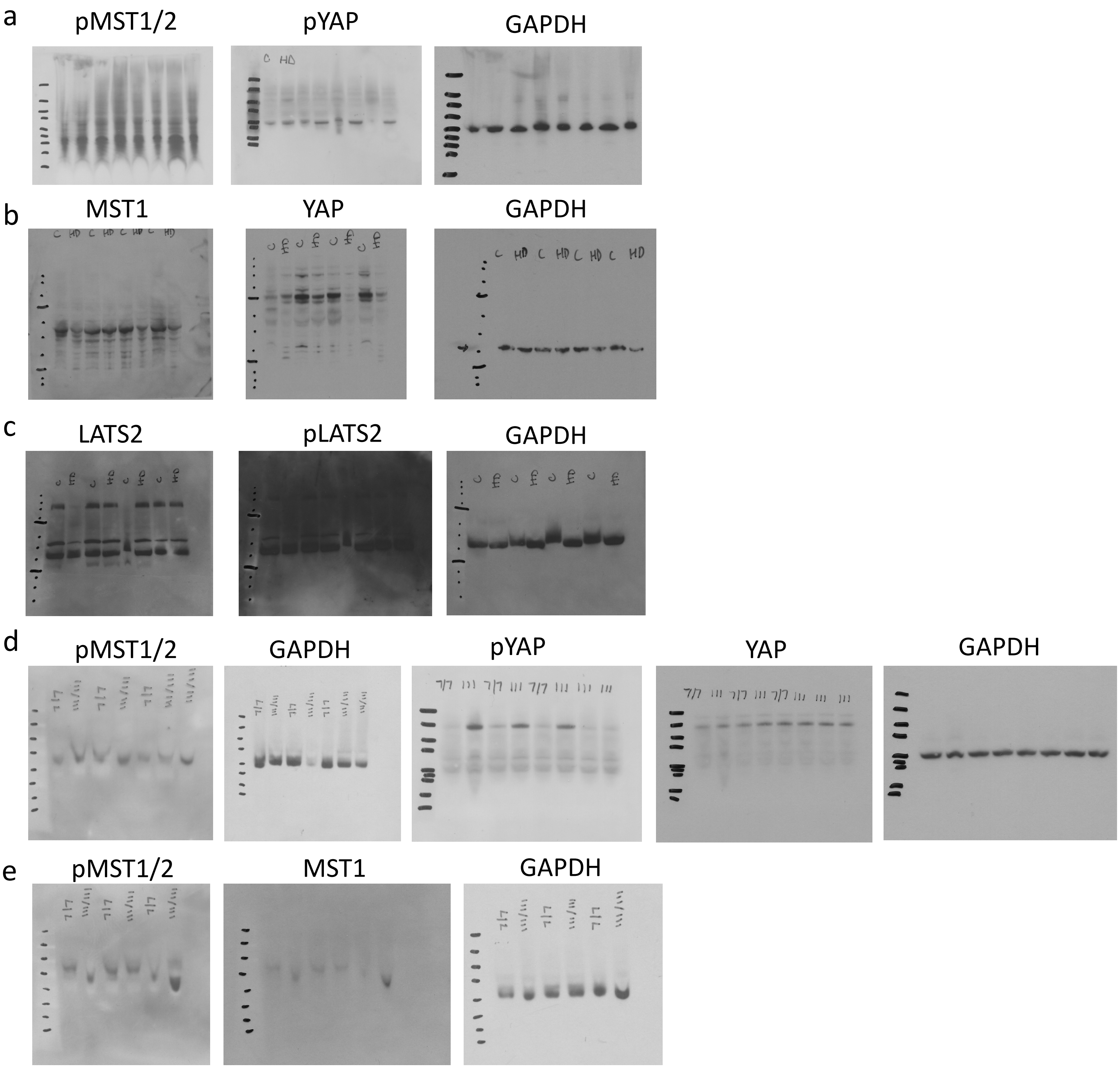
a
pMST1/2
pYAP
GAPDH
b
MST1
YAP
GAPDH
c
LATS2
pLATS2
GAPDH
d
pMST1/2
GAPDH
pYAP
YAP
GAPDH
e
pMST1/2
MST1
GAPDH

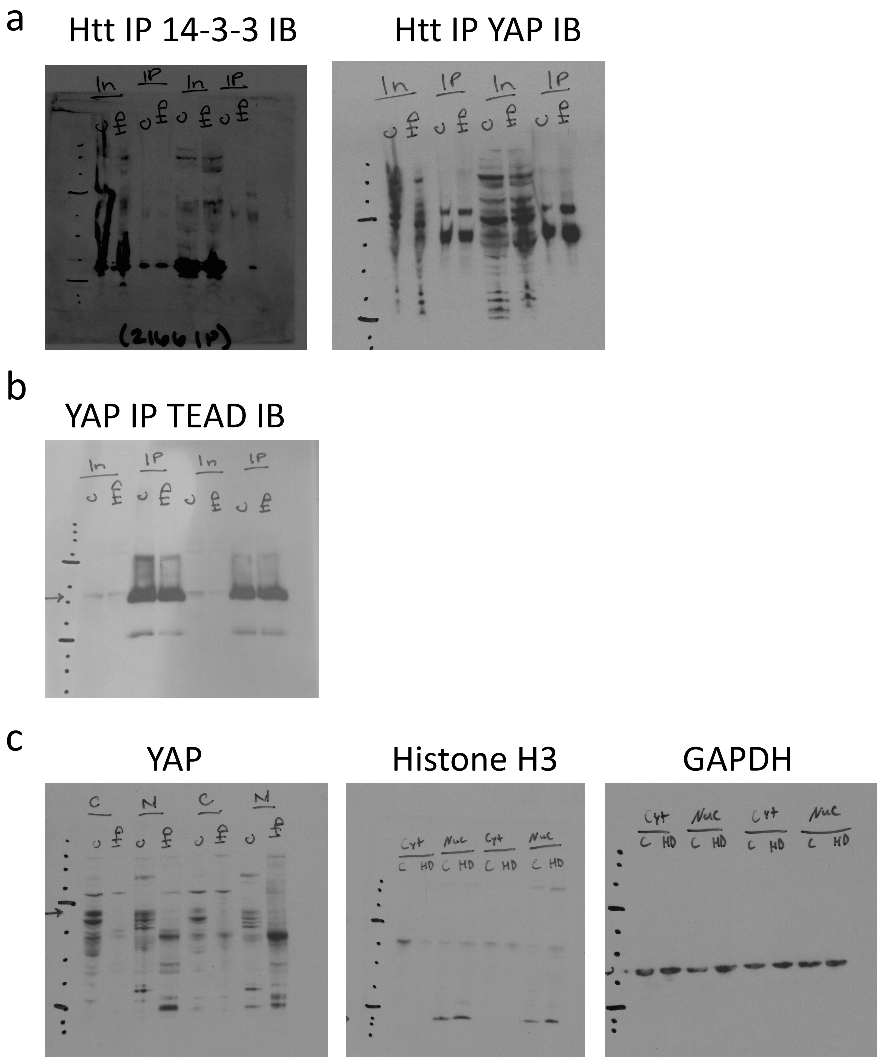
a
Htt IP 14-3-3 IB
Htt IP YAP IB
b
YAP IP TEAD IB
c
YAP
Histone H3
GAPDH

Supplementary Figure Legends

Figure S1. YAP, NeuN and DAPI cell counts in post-mortem human cortex

a) The total number of YAP+ cells were decreased (p=0.0044, Mann-Whitney *U* Test) in HD (black; n=10) compared to control (greyl n=7) human tissue. **b**) The total number of NeuN+ cells were not changed (p=0.1689, Mann-Whitney *U* Test) in HD compared to control human tissue. **c**) The total number of DAPI+ cells were increased (p=0.0172, Mann-Whitney *U* Test) in HD compared to control human tissue. Data are presented as median and min-max values. * p<0.05, **p<0.01.

Figure S2. YAP expression in GFAP+ cells in post-mortem human cortex

a) Representative images from unaffected control (top; n=7) and HD (bottom; n=10) human cortical tissue stained with YAP (green), GFAP (red), and DAPI (blue) (left to right). Merged images (far right) show overlap of YAP, GFAP and DAPI. Arrows highlight representative cells; scale bar = 50μM. **b**) The percentage of GFAP+ cells that were YAP+ (YAP/GFAP%) was not different (p>0.9999, Mann-Whitney *U* Test) in HD (black) compared to control (grey) human tissue. Data are presented as median and min-max values.

Figure S3. Decreased nuclear YAP in human HD cortex

a) Left, representative western blots for YAP (top) and histone H3 (bottom) using human cortex nuclear extracts from unaffected control (left; n=6) and HD (right; n=6) patients. Right, graph illustrating a decrease (p=0.0152, Mann-Whitney *U* Test) in nuclear YAP in HD (grey) compared to controls (black). **b**) Left, representative western blots for YAP (top) and GAPDH (bottom) using human cortex cytoplasmic extracts from unaffected control (left; n=6) and HD (right; n=6) patients. Right, graph illustrating no change (p=0.3095, Mann-Whitney *U* Test) in cytoplasmic YAP in HD (grey) compared to controls (black). Data are presented as median and min-max values. * p<0.05.

Figure S4. YAP subcellular localization in human iPSC-derived NSCs

a) Representative images from unaffected control (WT97) (top; n=6) and HD (HD4) (bottom; n=6) human iPSC-derived NSCs stained with YAP (green; left) and DAPI (blue; middle). Merged images (right) show overlap of YAP and DAPI. Scale bar = 25μM. **b**) Overall YAP intensity was not different (p>0.9999, Mann-Whitney *U* Test) between control (grey) and HD (black) NSCs. **c**) There was a trend towards a decrease in nuclear YAP signal (p=0.0931, Mann-Whitney *U* Test) in HD compared to control tissue. Data are presented as median and min-max values.

Figure S5. Uncropped western blots from Figure 3

Uncropped representative western blots from **a**) Figure 3a probed for pMST1/2, pYAP, and GAPDH (left to right); **b**) Figure 3c probed for MST1, YAP, and GAPDH (left to right); **c**) Figure 3e probed for LATS2, pLATS2, and GAPDH (left to right); **d**) Figure 3g probed for pMST1/2, GAPDH, pYAP, YAP, and GAPDH (left to right); **e**) Figure 3i probed for pMST1/2, MST1, and GAPDH (left to right).

Figure S6. Uncropped co-immunoprecipitation assay and western blots from Figures 4, 6, and S3.

Uncropped representative blots from **a**) Figure 4 co-immunoprecipitation assay Htt IP/14-3-3 IB and Htt IP/YAP IB (left to right); **b**) Figure 6 co-immunoprecipitation assay YAP IP/TEAD IB; **c**) Figure S3 western blot probed for YAP, Histone H3, and GAPDH (left to right).

Supplimentary Table 1. Hippo array gene list. * denotes significant differences in gene expression between control and HD cortex. MAPK10 is the only gene that was upregulated in HD as compared to control, indicated in red. * $p<0.05$, ** $p<0.01$, *** $p<0.0001$.

Gene Name	***Significant Change***
ACTG1	
AMOT	
AMOTL1	
AMOTL2	
CASP3	
CCNE1	
CCNE2	
CRB1	
CRB2	
CRB3	
CSNK1D	
CSNK1E	
DCHS1	
DCHS2	
DIAPH2	
DLG1	***
DVL2	
FAT1	*
FAT2	
FAT3	
FAT4	
FGX1	
GPC5	
HIPK2	*
HMCN1	
AJUBA	
LATS1	
LATS2	*
LIMD1	
LIX1L	**
LLGL1	

LLGL2	
LPP	
MAPK10	***
MEIS1	**
MOB1B	**
MOB1A	
MPDZ	
MPP5	
MST1	**
MYC	
NF2	***
NPHP4	
PARD3	
PARD6G	
POTEF	
PPP2CB	***
PPP2R1A	
PPP2R2D	
PRKCL	*
PRKCZ	
PTPN14	
RASSF2	***
RASSF4	
RERE	**
SAV1	**
SCRIB	
SMAD1	*
STK3	
STK4	*
TAOK1	
TAOK2	
TAOK3	
TAZ	
TEAD1	
TEAD2	
TEAD3	
TEAD4	
TJP1	
TJP2	

TP63	
TSHZ1	*
TSHZ2	
TSHZ3	
WNT1	
WTIP	
WWC1	
WWTR1	
YAP1	
YWHAB	
YWHAE	
YWHAQ	
YWHAZ	
ZDHHC18	
